# Structure/function of ATP sulfurylase domain of human 3′-phosphoadenosine 5′-phosphosulfate synthase (hPAPSS)

**DOI:** 10.1016/j.bbrep.2024.101892

**Published:** 2024-12-12

**Authors:** K.V. Venkatachalam, Dhiraj Sinha, Chris Soha, Rudi H. Ettrich

**Affiliations:** a College of Allopathic Medicine, USA; b Department of Radiation Oncology, UT Southwestern Medical Center, Dallas, TX, 75390, USA; c Health Professions Division, Nova Southeastern University, Ft. Lauderdale, FL, 33328, USA; d College of Biomedical Sciences, Larkin University, Miami, FL, 33169, USA; e Department of Cellular Biology & Pharmacology, Herbert Wertheim College of Medicine, Florida International University, Miami, FL, 33174, USA

## Abstract

3′-phosphoadenosine 5′-phosphosulfate (PAPS) is synthesized by PAPS synthase (PAPSS) in two steps. In the first step ATP sulfurylase (ATPS) transfers sulfate group onto adenylyl moiety of ATP to form adenosine 5′-phosphosulfate (APS) and PPi. APS-kinase (APSK) then transfers the gamma-phosphoryl from ATP onto 3′-OH of APS to form PAPS and ADP. Mutations of histidine's (H_425_/H_428_) of hPAPSS isoform1 knocked out ATPS and not APSK. *In silico* ATP binding and molecular dynamics experiments exhibited an unfavorable binding energy for mutant enzymes. Thus, requirements of H_425_NGH_428_ motif for ATPS is established. The N_426_ residue in various organisms is substituted with R. We mutated hPAPSS1 with basic residue K. The N_426_ to K_426_ (N–K) mutant exhibited slightly lower Km (3.7 mM) and higher Vmax (3X) for ATP compared to wildtype (WT, Km 4.3 mM). The Km for sulfate for N–K mutant was nearly same as WT but the Vmax was ∼4X higher for N–K. The catalytic efficiency (Vmax/Km) of N–K was ∼3 fold higher than WT. The full length hPAPSS1 evinced bimodal response against ATP, a paradigm that was deduced to be a trait of PAPSS that requires 2 mol of ATP/PAPS formed. This bimodal kinetics with ATP was lost when the N-terminal APSK was deleted from the C-terminal ATPS domain. The C-terminal domain contained ATPS activity, exhibited Km of 2.2 mM for ATP and Km of 0.53 mM for Sulfate and much higher catalytic efficiency compared to full length hPAPSS1. Thus, fused ATPS-APSK must be structurally and kinetically different than individual domains influenced by inter-domain residues.

## Highlights

• STRUCTURE/FUNCTION of human 3′-phosphoadenosine 5′-phosphosulfate (PAPS) hPAPS synthase.
• Importance of the HNGH motif in ATP binding of the STP sulfurylase domain.
• The role of two histidine residues in adenylyl transferase activity of the ATP sulfurylase of the hPAPSS.
• Gain of function of N to K mutant of ATP sulfurylase domain of hPAPSS and its effects.
• Discussion on two different modes of ATP use between the ATP sulfurylase and APS Kinase domains of hPAPSS.

## 1 Introduction

3′-Phosphoadenosine 5′-Phosphosulfate (PAPS) is the universal sulfuryl donor. PAPS is synthesized by bifunctional PAPS synthase (PAPSS) that contains N-terminal APS kinase (APSK) and carboxy terminal ATP sulfurylase (ATPS) domains [1]. First the carboxy terminal ATPS domain of hPAPSS1 (residues ∼220–623) condenses the sulfuryl group from inorganic sulfate on to adenylyl moiety of ATP to form **Adenosine 5′-Phospho Sulfate** (APS) and PPi. In the second step the 3′-hydroxyl of APS is phosphorylated by the amino terminal domain (residues ∼1–268) by APS kinase using ATP to form PAPS and ADP [2]. In humans there are three isoforms referred to as hPAPSS1, hPAPSS2a, hPAPSS2b [1]. hPAPSS1 and 2 are ∼77 % identical and the hPAPSS 2b differs from 2a by possessing an extra pentapeptide sequence GMALP [1]. All three hPAPSS isoforms contain typical N-terminal APSK domain and C-terminal ATPS domain deduced by linear amino acid sequence that contains conserved ATP binding motifs [1]. The exact regions that are necessary for APS-kinase and ATP sulfurylase domain is not clear for isoforms especially the 2a and 2b. However, previous study with hPAPSS1 indicated residues between 220 and 265 (referred to as linker sequence) is a must, for both APS kinase and ATP sulfurylase activities [2]. ATPS is a alpha-beta ATP bond splitting enzyme in contrast to the most common beta-gamma bond cleaving kinases. On the other hand, APSK is a beta-gamma phosphoanhydride ATP bond splitting enzyme that has the P-loop with a typical Walker A motif (GxxGxxK) [[4], [5], [6]]. The transfer of gamma-phosphoryl from ATP onto 3′-OH of APS is purported to require Mg_2_^+^ that interacts with residues of the D_87_GD_89_N phosphotransferase motif. Mutation of both aspartic residues (D_87_,D_89_) abolished APSK activity without any drastic consequences for ATPS activity [7]. Phosphoadenosine 5′-phosphosulfate (APS) binds to ATPS and APSK. In ATPS domain the APS is a product and for APSK it is a substrate. D_87_GD_89_N is absent in ATPS but present in APSK. Mutation of both aspartic residues (D_87_,D_89_) into alanine did not hamper the ATPS but abolished APSK activity [7].

ATP sulfurylase lacks the GxxGxxK motif for ATP binding, instead it contains conserved H_425_NGH_428_ [3] residues coined here as Venk motif. Previously, site selected mutations of the H_425_ and H_428_ single mutant into alanine completely knocked out the ATPS activity [3]. ATPS being a adenylyl-sulfotransferase, transfers sulfate group onto adenylyl moiety of ATP which then releases the products APS and PPi. The released PPi is hydrolyzed by ubiquitous pyrophosphatase into 2Pi. This drives the thermodynamically unfavorable ATPS reaction forward. In contrast most kinases (gamma-phosphoryl transferases) are beta-gamma bond splitting enzymes that binds ATP and drives the reactions thermodynamically in the forward direction [1,2]. Whereas ATPS enzyme is unfavorable in the forward direction, is driven forward with the help of separate pyrophosphatase enzyme [1,2]. Thus, PAPSS is the only bifunctional enzyme that we know of that uses ATP in two different manners. ATPS domain is a alpha-beta phosphoanhydride bond cleaving type and APSK is a beta-gamma phosphoanhydride bond cleaving activity domain. In this paper we report that the dual mutation of H_425_A-H_428_A makes the ATP binding nearly impossible with high energy required compared to wild type (WT). In addition, this is the first report to show the advantage of basic residue at position 426. In hPAPSS1, 2a and 2b the residue 426 is an asparagine (N). When N426 is mutated to K it improves the catalytic efficiency of hPAPSS1 by 3–4 folds. ATPS from various organisms *Riftia symbiont*, *Saccharomyces cerevisiae*, *Penicillium Chrysogenum*, *Aquifix aeolicus*, and *Thermus thermophilus* contained R instead of N in the HNGH region of hPAPSS [8]. The *in silico* binding kinetics of N_426_–K mutant of hPAPSS1 exhibit much less energy than WT for ATP binding. In this paper we also report the kinetics of individual C-terminal domain (polypeptide 220–623) and N_426_–K mutant which are quite different and fascinating.

## 2 Methods

Materials-For enzyme assays radioactive inorganic [^35^S]SO_4_ (1300 Ci/mmol), and for DNA sequencing [α-^35^S]ATP were purchased from NEN Life Science Products. From the companies of Life Technologies, Inc. and Gene Probe Technology (Gaithersburg, MD).oligonucleotides were purchased. From U. S. Biochemical Corp. version-2 sequencing kit was obtained. Agarose was purchased from FMC BioProducts (Rockland, ME) Polyethyleneimine cellulose (PEI)-TLC plates were purchased from Merck. Site-directed mutagenesis kit was obtained from Stratagene (La Jolla, CA). x-ray film was purchased from Eastman Kodak Co.

### 2.1 Site-selected mutagenesis

The conserved motif (H_425_NGH_428_)) located in the COOH-terminal region of human PAPS synthase was used for site selected mutagenesis experiments. Amino acid substitutions were carried out corresponding to the H_425_NGH_428_ sequence. Using the quik change mutagenesis kit from Strategene, mutations were performed by following instructions from the manufacturer. For example, oligonucleotides containing the respective base substitutions corresponding to the selected mutated codons were synthesized. Mutations were performed by PCR using the wild type hPAPS synthase expression vector (plasmid pET-19b) as template and substituted oligonucleotides as primer. Pfu DNA polymerase was used for DNA amplification. PCR consisted of 12 cycles of denaturation at 94 °C for 30 s, annealing at 55 °C for 1 min, and synthesis at 68 °C for 12 min. Once the PCR is done, *Dpn*I restriction enzyme was used for degrading the methylated parent template plasmid. Transformation was performed using XL1-Blue supercompetent cells and circular dsDNA. Colonies were isolated and used for fetching plasmids that possessed the correct sequence which was confirmed by DNA sequencing. Confirmed plasmids with mutations were used for transformation by using protease deficient bacteria (pLyz BL21-DE3). Further, plasmids were isolated and the sequence was verified for the presence of the appropriate mutation.

### 2.2 Overexpression of human PAPS synthase and mutant constructs

Full-length hPAPS synthase (GenBankTM accession number U53447) (wildtype) and mutant hPAPS synthase plasmids were PCR amplified using primers containing *Bam*HI restriction sites. They were then cloned into *Bam*HI digested pET-19b vectors that contained proprietary 122 base pair *Nco*I-*Nde*I cassette (Veritas, Potomac, MD). The vector contained sequences for calmodulin binding site of calcineurin followed downstream by histidine tag and an enterokinase cleavage site. Transformation into competent DH-5α *Escherichia coli* was done as before using CaCl_2_ [2]. Colonies were isolated, miniprepped, and plasmids were sequenced for correct orientation of the initiator codon with respect to the T7 promoter sequence. Transformation was performed with expression host cells from Stratagene (BL21-DE3 plyz) using the pET-19b vectors containing the correct colned inserts with proper orientation.

### 2.3 Preparation of bacterial cell extracts

Bacteria was grown in LB broth containing ampicillin. Once the turbidity reached to A595 nm of 0.5, IPTG (1 mM) was added to the culture for inducing protein expression for 3h. After 3 h cells were centifuged to obtain the pellet. Then, 150 ml of lysis buffer (20 mM Tris-HCl, pH 7.5, containing 50 mM KCl, 1 mM dithiothreitol, 10 % glycerol, and 1.2 mg/ml lysozyme) was used for resuspending the pellet. Resuspended cells were transferred into centrifuge tubes. The original tube was then washed with100 ml of lysis buffer and the wash was added to the original cell suspension. Cell lysis was carried out at 25 °C for 7 min. The lysates were then centrifuged for 15 min at 10,000 g at 4 °C. The clear supernatants were purified by Ni + coulumn. Further purification was done by gel filtration. The purified preparations were used for enzyme assays.

### 2.4 PAPS synthase assay

Reactions were performed in a total volume of 10 μl. The enzyme reaction consisted 3 μl of reaction buffer (150 mM Tris-HCl, pH 8.0, 50 mM KCl, 15 mM MgCl_2_, 3 mM EDTA, and 45 mM dithiothreitol), 1 μl of 50 mM ATP, 3 μl of sample. The reaction was strated by adding 3 μl of inorganic [^35^S]SO_4_ (∼3.4 μCi). Reactions were carried out for 30 min at 37 °C. The reaction was stopped by placing the tubes in boiling water for 5 min. After cooling 1 μl was placed onto PEI-TLC plates. The PEI-TLC plates were chromatograped using 0.9 M LiCl solvent. Dried PEI-TLC plates were exposed overnight on to x-ray film.

The respective radioactive spots corresponding to PAPS, APS, and SO_4_ were cut out, and the liquid scintillation activity was measured by standard counts per minutes (cpm).

## 3 Computational methodology

Given that the crystal structures of human PAPSS1 bound to various ligands have been resolved by X-ray diffraction to a high resolution of up to 1.75 Å (PDB entries: 1x6v, 1xjq, 1xnj, 2qjf), we can utilize atomistic molecular dynamics simulations and ligand-docking techniques to gain deeper insights into the structural and functional roles of specific residues within the conserved HXGH motif, which forms part of the substrate binding pocket in the ATP sulfurylase domain. In the initial phase of this work, we used homology modeling with YASARA [9,10] to address missing loop regions, conduct *in silico* mutagenesis, and prepare the ATP-Sulfurylase domain (ATPS) of human PAPSS1 for molecular docking studies. We used a 2.2 Å resolution crystal structure of the ATP-Sulfurylase domain of human PAPSS1, complexed with Adenosine-5′-Phosphosulfate, obtained from the Protein Data Bank (PDB ID: 2qjf). The structure comprises two identical chains, labeled A and B. While both chains were included in all docking experiments, chain B was specifically chosen for positioning the simulation cell and serving as the receptor site for ligand docking.

To prepare the structure for docking, all ligands, phosphate, and water molecules were removed, and hydrogen atoms were added following the standard procedure in YASARA [9] and then the entire structure underwent energy minimization. The Mg-ATP ligand was prepared using structures from the Protein Data Bank, with YASARA's AutoSMILES feature employed for automatic force field parameter assignment. AutoSMILES utilizes SMILES strings to identify known molecules and applies the AM1BCC and GAFF [11,12]. (General AMBER force field) methods for molecules not recognized. AM1BCC charges were further refined by incorporating known RESP charges of similar molecular fragments and by calculating semi-empirical AM1 Mulliken point charges [13], which involved geometry optimization using the COSMO solvation model [14].

For molecular docking, YASARA employs AutoDock-Vina [15], which uses an iterated local search global optimizer algorithm to predict binding poses and a semi-empirical scoring function to evaluate and rank them. To ensure an unbiased search for binding pockets, a simulation cell was defined to extend at least 5 Å beyond all atoms of domain B. We conducted 100 docking runs in the global search mode of AutoDock Vina, as implemented in YASARA, to dock the ligands to the ATP-sulfurylase domain of human PAPSS1.

The resulting conformations were grouped into clusters, each differing by a minimum heavy atom RMSD of 5.0 Å. From each cluster, the conformation with the highest binding energy was selected as the representative structure. Throughout the docking process, the protein structure was maintained in a fixed position, while the ligand was permitted full flexibility to explore potential binding poses.

To validate our docking methodology, we initially re-docked Adenosine-5′-Phosphosulfate to verify that the binding site was accurately predicted as per the crystallographic data. Following this, MgATP was docked, with the conformation exhibiting the highest binding affinity selected as the representative structure within the cluster.

We also performed *in silico* mutagenesis to generate single-point mutations (G427A and N426K) and a double mutant (H425A-H428A). These mutations were introduced by substituting the respective side chains within YASARA, followed by a brief 100 ps energy minimization, where non-mutated residues were constrained to avoid local structural distortions. These mutations were applied to each monomer of the ATPS domain of human PAPSS1, and energy minimization was conducted using the Amber 03 force field [12]. to maintain consistency with the force field used during the docking studies.

This detailed approach enables us to examine the structural and functional effects of these specific mutations in the ATPS domain. To analyze the system's dynamics, classical molecular dynamics (MD) simulations were performed with Gromacs version 4.6.5 [16], utilizing the AMBER99SB force field [11,17]. The system was placed in a cubic simulation box with dimensions of 10 × 10 × 10 nm and solvated using the TIP3P explicit water model [18,19]. To maintain electrostatic neutrality, Na + ions were added to replace randomly selected water molecules. The system then underwent steepest descent energy minimization with a maximum step size of 0.05 Å to address unfavorable van der Waals interactions in the initial configuration. Long-range electrostatic interactions were calculated using the Particle Mesh Ewald (PME) method with a 10 Å cutoff distance, and bond lengths were constrained with the LINCS algorithm employing a fourth-order expansion [20].

The parameterization of ATP ligands was carried out using the standard RESP procedure in Antechamber, with the partial charges for free Mg2+ ligands obtained from HF/6-31G∗ calculations in Gaussian03 [21]. Histidine residues were modeled with double protonation at the ND1 and NE2 positions, whereas arginine and lysine residues were protonated according to Gromacs default settings.

After energy minimization, each of the six systems was equilibrated by restraining the heavy atoms under constant temperature conditions with the V-rescale thermostat [22] at 300K for 1 ns. This was followed by pressure equilibration using the Parrinello-Rahman barostat [23] for an additional 1 ns at each temperature. Initial velocities were assigned according to a Maxwell-Boltzmann distribution at the corresponding temperature, and neighbor lists were updated every 10 fs using a group-based cutoff scheme. The minimized and equilibrated configurations were used as the starting points for all subsequent unrestrained MD simulations, which were performed for 100 ns each in the isothermal-isobaric (NPT) ensemble.

The Molecular Mechanics/Poisson-Boltzmann Surface Area (MM/PBSA) method, implemented in Gromacs as g_mmpbsa [24], was used to calculate the receptor-ligand binding free energy from the trajectories. Each trajectory was reduced to 500 snapshots, and the g_mmpbsa tool was utilized with default settings for the calculations.

Figures were generated using Yasara and rendered with POVRay (www.povray.org). Subsequent editing and labeling were completed using GIMP 2.10.18. (www.gimp.org).

## 4 Results

### 4.1 Nucleophilic attack of ATP

**Fig. 1:**
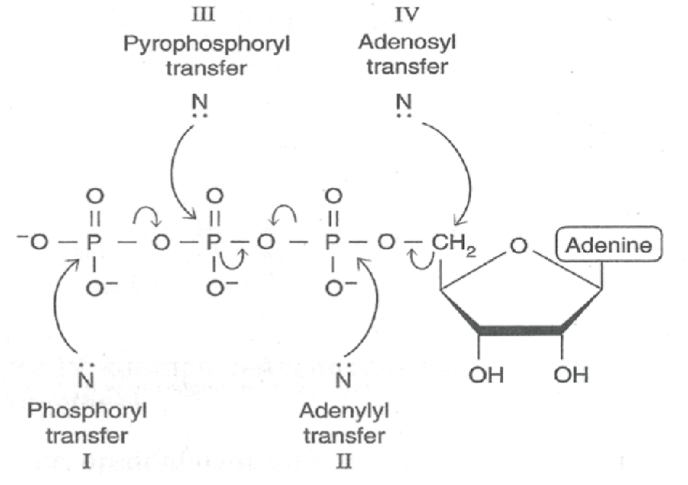
Mechanism of ATP use in various reaction types. ATP sulfurylase is a adenylyl transferase (Type II) and APS kinase is a Type I phosphoryl transferase.

ATP can possibly be reacted nucleophilically in four different ways. The possible nucleophilic attack of the molecule is described in Fig. 1.

**Fig. 2:**
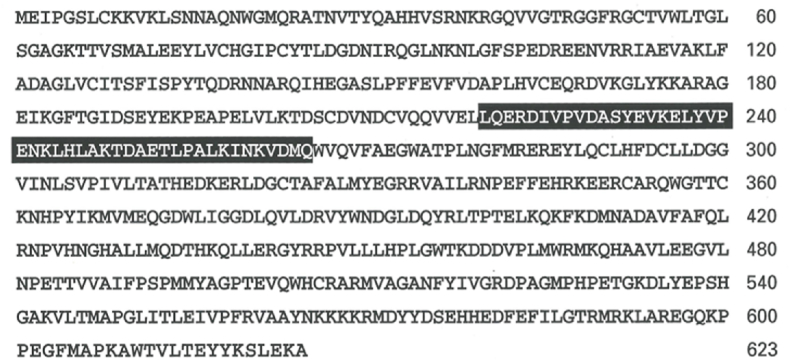
Linear amino acid sequence of hPAPSS1. Residues ∼1–265 encompasses APSK domain and residues ∼265–623 encompasses ATPS activity domain. The highlighted residues are common between two domains and mandatory for bifunctional (ATPS-APSK) activities of hPAPSS.

Most of the kinases are beta-gamma hydrolases. APS kinase is a beta-gamma hydrolase that splits the phosphoryl and transfers it to the recipient molecule that gets phosphorylated. In contrast the nucleophilic attack on alpha phosphoryl of ATP releases pyrophosphate (PPi) as the by product and the adenylyl portion gets added onto the attacking nucleophile. ATP sulfurylase binds inorganic sulfate and the sulfate oxy anion reacts with alpha-phosphoryl of ATP to form 5′-phosphoadenosine phosphosulfate (APS) and PPi. Thus, as classified in Fig. 1, ATPS falls under the category of adenylyl transfer (type II) reaction. In mammals ATP sulfurylase and APS kinase are fused gene products. Human PAPSS1 (hPAPSS1) contains NH_2_-terminal APS kinase domain and COOH-terminal ATP sulfurylase domain connected by a common linker region (residues 220–265) as shown in Fig. 2.

**Fig. 3:**
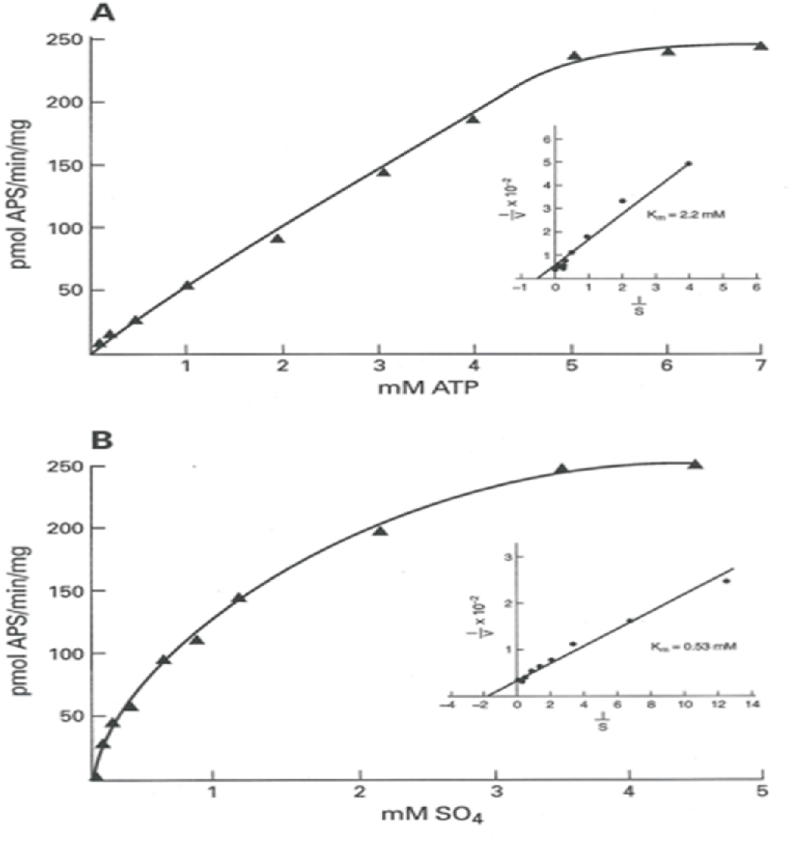
COOH-terminal (aa 220–623) ATPS domain activity. Fig. 3A shows increasing concentration of ATP plotted against velocity (pmol APS formed/min/mg). The corresponding inset is the Lineweaver-Burk plot of 1/s vs 1/v showing the Km value of 2.2 mM for ATP. Fig. 3B shows increasing concentration of Sulfate plotted against velocity (pmol of APS formed/min/mg). The corresponding inset is the Lineweaver-Burk plot of 1/s vs 1/v showing the Km value of 0.53 mM for sulfate.

Without the linker region both APSK and ATPS activities are negligible [1,2]. The COOH-terminal domain constructed by including residues 220–623 of hPAPSS1 was cloned, expressed, purified and characterized for incresing substrates of ATP with fixed sulfate concentration (Fig. 3A) and increasing ATP with fixed sulfate concentration (Fig. 3B). Line-Weaver Burk reciprocal plot (insets) indicates Km of 2.2 mM for ATP and 0.53 mM for sulfate.

**Fig. 4:**
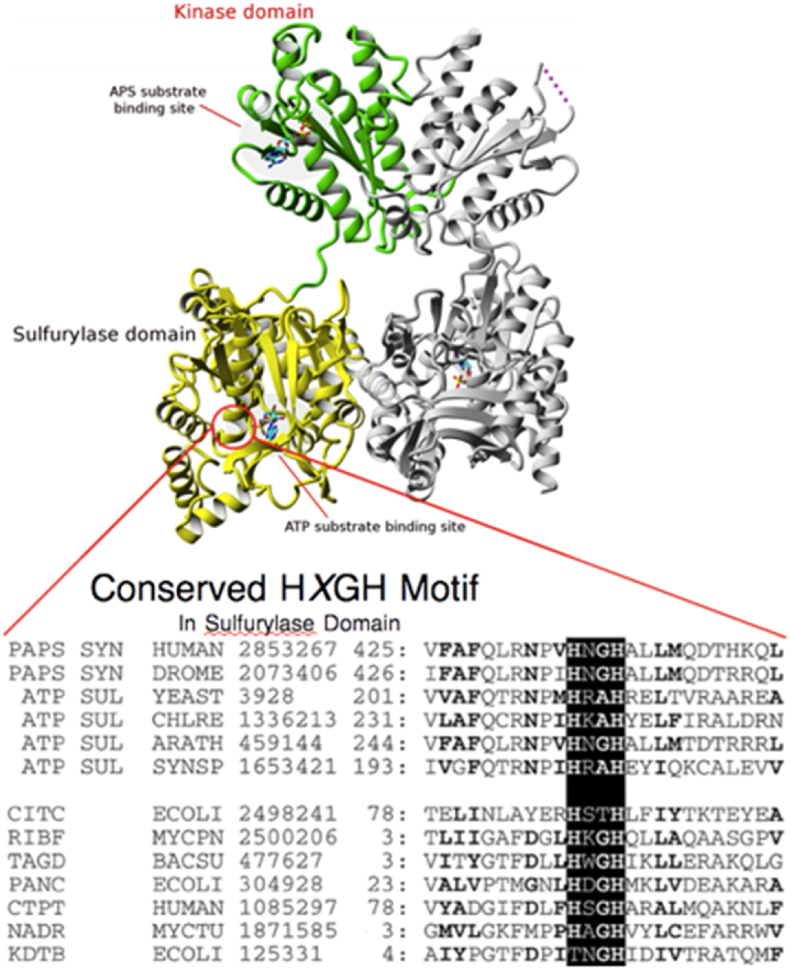
HPAPSS1 structure. The top ribbon diagram represents the dimeric structure of hPAPSS. Top Green and Silver ribbon diagram depicts APSK structure. The green ribbon structure also shows APS substrate (in red color) bound to it. The bottom yellow and silver ribbon diagram represents dimeric domain of ATPS. The circled part on the yellow ribbon possesses sequences that contains conserved HXGH motif.

ATPS being an alpha-beta ATP hydrolase doesn't contain typical Walker motif (GxxGxxK) as seen with APSK. APSK is a typical beta-gamma hydrolase which contains canonical Walker-A motif and ATPS instead contains conserved HNxH motif as shown in Fig. 4 below.

**Fig. 5:**
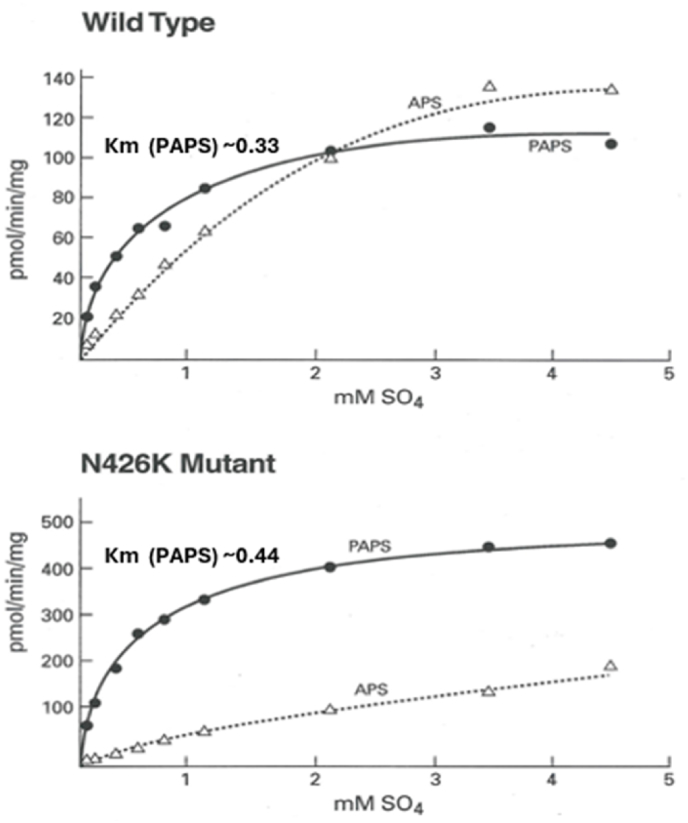
Increasing substrate concentration of sulfate versus reaction velocity of APS formed by full length WT (5A) and N_426_K mutant (5B) of hPAPSS1. Fig. 5A and B shows increasing concentration of sulfate plotted against velocity (pmol APS and PAPS formed/min/mg). Overall PAPS formed in WT and N_426_K mutant exhibited typical hyperbolic Michaelis-Menton kinetics. However the APS formed in WT reached saturation, N_426_K mutant was linear.

The full length construct containing 1–623 amino acid residues of hPAPSS1 cloned, expressed purified and characterized. The kinetics with various sulfate concentrations are represented below for wild type (WT) top panel (Fig. 5A). As can be seen for overall PAPS production (ATPS + APSK combined activities) hPAPSS1 shows typical Michaelis-Menton kinetics. The APS formation by ATPS trails APSK product PAPS at a slower rate. The bottom panel (Fig. 5B) represents the kinetics exhibited by N_426_K mutant. As compared to the WT, Km for overall activity of PAPS formation (ATPS + APSK combined activities) for N_426_K mutant was ∼4X higher whereas the Km value towards sulfate was similar to that of WT.

**Fig. 6:**
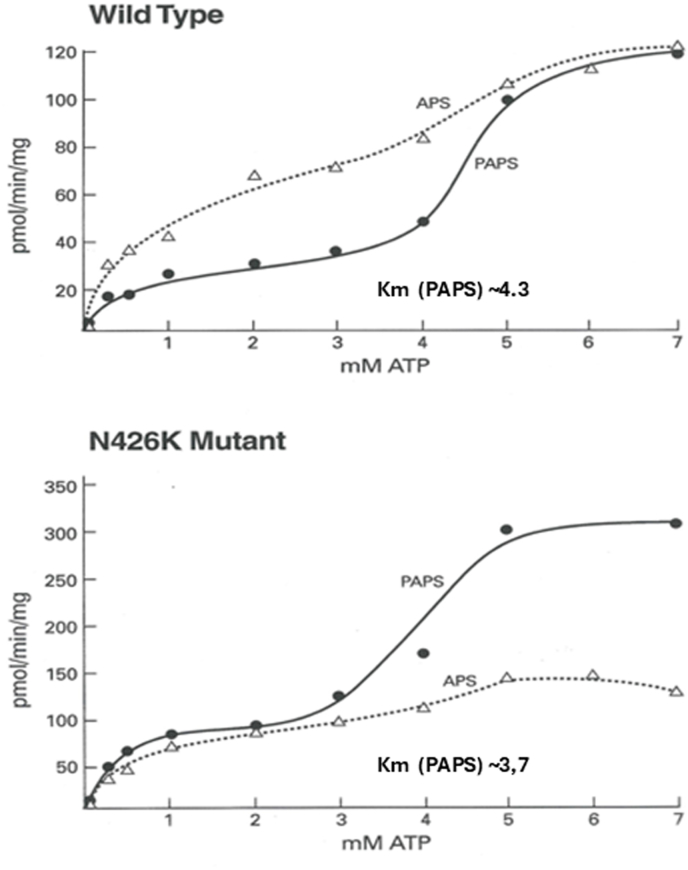
Kinetics of full length WT and N_426_K mutant enzymes against ATP. The overall PAPS formed exhibited bimodal curve evinced both in WT and N_426_K.

In Fig. 6A (the top panel) shows the response of WT enzyme towards varying concentration of ATP. The PAPS formation (solid circle) exhibits biphasic curve with first phase becoming flattening between 3 and 4 mM ATP and the second phase plateaus ∼ between 6 and 7 mM ATP with a highest Vmax reaching up to ∼120 pmol/min/mg. The N_426_K mutant enzyme (Fig. 6B) also exhibits bimodal response towards ATP however the Vmax was significantly higher reaching up to 300 pmol/min/mg which is ∼3 fold higher than WT. The intermediate APS accumulated was nearly similar between WT and N_426_K reaching up to ∼120 pmol/min/mg.

Since hPAPSS exhibited biphasic response towards ATP a half maximal estimate was determined to represent an approximate Km value. WT showed K0.5 of 4.3 mM and N_426_K mutant showed 3.7 mM, Km value that is slightly lower for N_426_K towards ATP. Contrary to ATP response, both WT and N_426_K mutant exhibited typical Michalis-Menton kinetics (a hyperbolic response) with a calculated Km (Lineweaver-Burk plot inset) of 0.33 mM for WT and 0.44 mM for N_426_K towards sulfate. The Vmax for N_426_K was 3–4 fold higher for ATP and sulfate compared to WT. The overall catalytic efficiency (Vmax/Km) was about 3 folds higher for N_426_K compared to WT (Table 1).

**Table 1:** Kinetic parameters exhibited by WT and N_426_K towards ATP and inorganic sulfate.

Construct	K_0.5_ ATP mM (half maximal estimate)	Km SO_4_ mM	Vmax ATP pmol/min/mg	Vmax SO_4_ pmol/min/mg	Vmax/K_0.5_ ATP	Vmax/Km SO_4_
Wild Type	4.3	0.33	120	118	28	357
N426K	3.7	0.44	310	470	84	1068

### 4.2 Binding of ATP and sulfate on the active site of hPAPSS1: *in silico* studies

**Fig. 7:**
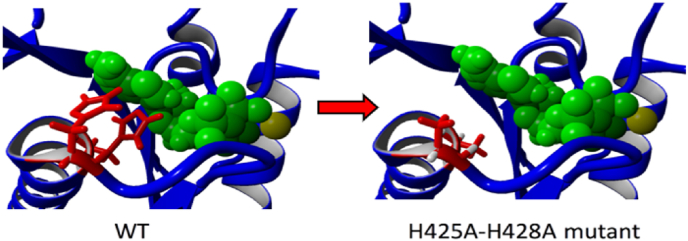
ATP docked into the binding pocket of the sulfurylase domain of PAPSS1. ATP is depicted as a ball and stick model in green. The histidine residues H425 and H428 and their respective mutations to Alanine are shown as a stick model in red, Left panel: WT, right panel:H425A-H428A double mutant.

Using high-resolution crystal structures of hPAPSS1 domains complexes of wild-type and mutant hPAPSS1 with bound ATP were created and molecular dynamics simulations were performed to describe movements of the atoms over time. As can be seen in Fig. 7 in WT the two histidine's (H_425_ and H_428_) side chain imidazole ring exhibited pi-pi interaction with anti-oriented purine ring structure of ATP and the overall ATP binding energy for hPAPSS1 as calculated by MM/PBSA is ∼ -40 kJ/Mol (Table 2), indicating good binding of ATP for the WT active site pocket. In contrast, when the two histidine's (H_425_ and H_428_) were replaced with alanine the R group methyl is not in close proximity for closer/fine interactions. Table 2 describes the overall binding energy differences between WT hPAPSS1 and mutants (N_426_–K, G_427_-A, and H_428_-A-H_425_-A) of the HNGH motif. As can be seen the double mutant exhibited an unfavorable binding energy for ATP. The ATP molecule which was artificially docked into the binding pocket to measure the binding energy thus would probably not stay long enough in the binding pocket, making the reaction thermodynamically impossible to take place during the intracellular time needed. The N426K mutant shows an even more favorable binding energy (−70 kJ/Mol) than WT, while the G-A mutant exhibited an unfavorable binding energy (218 kJ/Mol) for ATP. This correlates well with the experimental findings that even single mutants of the two histidine render the enzyme into a totally inactive enzyme as measured by overall PAPS assay [3].

**Table 2:** MM/PBSA binding energies of MgATP in complex with hPAPSS1.

	WT (ATP) kJ/mol	G427A (ATP) kJ/mol	N426K (ATP) kJ/mol	H425A_ H428A (ATP) kJ/mol
MM-PBSA Binding energy	−40	218	−70	528

**Fig. 8:**
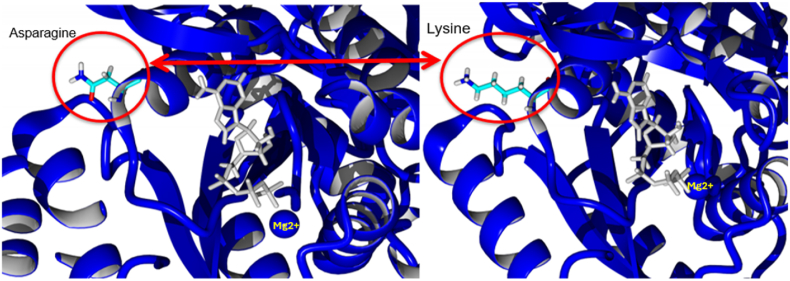
In Fig. 8 shown below the active site pocket of WT (with asparagine at position 426) is compared with the mutant enzyme replaced with basic residue lysine. Position of Mg-ATP after 100 ns of molecular dynamics simulation, left panel: WT, right panel: N426K mutant. The magnesium ion is depicted as a blue sphere while ATP is shown in grey as a stick model.

In WT the ATP (depicted with silver sticks) is closer to Mg_2_^+^ (blue ball with Yellow Font on top). Whereas in N426K mutant, after 100 ns of MD simulation the ATP phosphates were found to be significantly closer with looped structure forming even better contacts with cationic (Mg_2_^+^) (Blue Ball with Yellow Font on Top). From this we predict that lysyl-ε−amino group positive charge neutralizes the repulsive forces of anionic charges and stabilizes the active site complex for nucleophilic attack by inorganic sulfate that can happen with ease. (see Fig. 8).

**Fig. 9:**
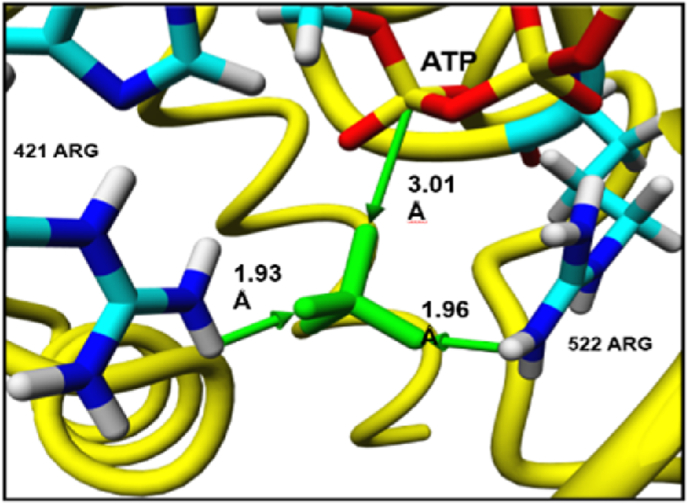
Sulfate docked into the ATP-PAPSS1 complex. Sulfate is depicted in green as a stick model.

To further test the above idea, docking of inorganic sulfate was done using autodock VINA. As can be seen in Fig. 9 there are two arginine's (proximal R421 and distal R522) that are in closer proximity at a respective distance of 1.93 and 1.96 Å. Out of the four oxygens of the inorganic sulfate, two oxyanions normally bears electro negative charges. One oxyanion had to engage in nucleophilic attack and the other neutralized. As can be seen in Fig. 9, one of the oxyanions is about 3.01 Å distance perhaps poised for nucleophilic attack with alpha phosphoryl group of ATP.

## 5 Discussion

PAPSS synthase (PAPSS) is a bifunctional enzyme in many organisms [1,25,26]. PAPSS produces 3′-phosphoadenosine 5′-phospho sulfate (PAPS) in two steps. In the first step inorganic sulfate reacts with ATP to form PPi and adenosine 5′-phosphosulfate catalyzed by ATP sulfurylase (ATPS) [1]. In the second step APS kinase (APSK) uses APS and ATP to form 3′-phosphoadenosine 5′-phosphosulfate (PAPS) and ADP [1]. PAPS is the universal sulfuryl donor. Sulfotransferases use activated sulfuryl group from PAPS to transfer, onto recipient hydroxyl containing molecule to form sulfate-ester [27] The sulfurylated molecule receives a negative charge that changes the physicochemical properties of the molecule [27]. Thus, PAPS production and its regulation is crucial for all biological sulfonation and consequent cell integrity and metabolism. We used human PAPSS 1 (hPAPSS1) as a model system to study the structure function. As reported earlier, the hPAPSS1 has two domain activities ATPS and APSK. Previous study delineated the regions of ATPS activity domain and APSK [2]. As shown in Fig. 1 the ATP molecule can participate in four different ways. Most of the serine/threonine and tyrosine kinases are type I that transfers gamma-phosphoryl of ATP on to the recipient amino acid residues that gets phosphorylated [28,29]. Many small molecules, one for example glucose gets phosphorylated inside the cell by hexokinases [30] using ATP to form glucose 6-phosphate which are also Type I phosphoryl transferase. ATP sulfurylase (ATPS) is a type II that transfers adenylyl portion of ATP onto the recipient sulfate molecule to form APS and PPi [3]. In contrast to ATPS, CTP:phosphocholine cytidylyltransferase uses CTP to transfer cytidyl group on to phosphocholine to form CDP-choline [31]. Though type III is a possible mechanism in biological system to use ATP as the moiety for ADP transfer, thus far no known report is available to support the existence of such transferase (Venkatachalam proposed). Nonetheless, ADP transfer is a common occurrence in biological system in wide variety of organisms, as for example histone ADP ribosylation which use NAD + as the ADP donor instead of ATP [32]. The type IV transfer involves the transfer of adenosyl group from ATP onto a recipient molecule. Methionine adenosyl transferase (MAT) is the only enzyme that falls on to the type IV category that transfers the adenosyl group from ATP on to sulfur of methionine to form the universal methyl group donor *s*-adenosylmethionine (SAM) [33]. In MAT catalyzed reaction it is quite interesting that all three phosphates are released as PPi and Pi [33]. Thus, the interplay of phosphate and sulfur metabolism is well established across organisms [34,35]. PAPSS is the one and only enzyme that is known thus far to use ATP in two different mechanisms. ATPS domain activity of PAPSS is a type II and APSK domain of PAPSS is a type I transfer entities (Fig. 1). Type I phosphoryl transfer seen with protein and small molecule kinases use typical Walker motif [4,5]. APSK and putative APSK domain of all PAPSS possess typical Walker A motif GxxGxxK [1]. In all three hPAPSS (1, 2a and 2b) the ATP binding P-loop motif contains conserved GLSGAGKT on the N-terminus. In hPAPSS1 G_59_LSGAGKT_66_ is between residues 59–66 [2,8]. In hPAPSS2a and 2b G_49_LSGAGKT_56_ is between residues 49–56 [2,8]. ATPS being a type II transfer type doesn't contain P-loop [6] with Walker motif [[4], [5], [6]] instead it contains HNxH motif [1,3]. In all hPAPSS it is HNGH residues (Venk motif) that is purported to bind ATP. In hPAPSS1 “HNGH” is between residues 425–428. In hPAPSS2a it is between residues 415–418 and in hPAPSS2b it is between residues 420–423. It is interesting that the hPAPSS isoforms are about 5–10 residues apart from each other on the linear primary sequence. In this paper we present the experimental studies on the mutational effects of the HNGH motif of the hPAPSS1. In addition, the *in silico* studies are performed on hPAPSS1 on the HNGH motif. With hPAPSS1 the residues H_425_NGH_428_ were experimentally studied for kinetic parameters. The N_426_K mutant exhibited higher Vmax and catalytic efficiency (Vmax/Km). The residue next to histidine in many organisms is substituted with arginine and the physiological significance is unknown. Perhaps in organisms which reduce sulfate to sulfide and subsequent reaction of hydrogen sulfide with O-acetylserine to form cysteine and homocysteine followed by methionine synthesis may require higher amounts of PAPS synthesized [36,37]. The reductive pathway of activated sulfate PAPS into sulfite by PAPS reductase is in high demand in plants [38,39] and is totally absent in mammals. This warrants a less active PAPS synthase since the product PAPS in mammals is used only for limited and specific sulfotransferase reactions [27]. Thus, we speculate that the conversion of N into K of WT in hPAPSS leads to gain of function. In other words, nature had done the mutation to convert a more basic residue (R) seen in certain organisms (bacteria, yeast etc.) into a neutral residue as seen with mammals. This change perhaps evolved according to the PAPS demands, dictated by the respective organism. The mutation of both histidine's (H_425_ and H_428_) into alanine completely abolished its activity. The G_427_ to A mutation resulted in loss of activity in the forward direction of PAPS formation and the backward reaction of PPi combining with APS to form ATP and sulfate assessed by radioactive ^35^SO4 formation was much faster a paradox that we can't explain with available data (data not reported). When the C-terminus (residues 220–623) was separated from the crucial parts (1–219) of APSK domain it exhibited typical Michalis-Menton (MM) kinetics with hyperbolic response with both ATP and sulfate. On the other hand, the WT full length (residues 1–623) exhibited a bimodal/biphasic response with ATP and typical M-M response with sulfate. This response is true with full length N426K mutant as well exhibiting a bimodal/biphasic response with ATP and typical M-M response with sulfate. Thus, in fused gene product (ATPS-APSK) of PAPSS the two domains are coordinately influencing each other's activities in overall PAPS synthesis controlled perhaps by ATP availability. Our experimental data is fully supported by *in silico* data in terms of ATP binding energies. That is the H_425_-A-H_428_-A double mutant possessed no activity [1,2] and it is corroborated by high energy required for ATP binding presented in the results section. Thus, the double mutant is highly thermodynamically unfavorable to drive the reaction. We tested this hypothesis with conserved HNGH motif of hPAPSS2b H_420_-A-H_423_-A double mutant. The mutation of two histidine's (H_420_–H_423)_ drastically increased binding energy for ATP a response that was less severe with hPAPSS2b compared to hPAPSS1. This means the ATP binding pocket is less stringent with hPAPSS2b compared to hPAPSS1 for ATP binding and in part explains the two isoforms are structurally different which reflects in the functional differences. Finally, when we looked at the active site binding of sulfate the reactive oxy anions are neutralized by two arginine's (R_421_ and R_522_). This makes the sulfate fixed nucleophile with one reactive oxyanion poised for nucleophilic attack of the alpha-phosphoryl of ATP to form APS and PPi. In N_426_–K mutant the loop structure in the active site pocket is drastically different making the sulfate closer to ATP than seen with WT which additionally explains the higher activity exhibited by the N_426_–K mutant. In addition, the tradeoff between substrate binding (a parameter of Km) and the product release is an orchestrated balanced event in that both WT and N_426_–K mutant exhibits similar Km for sulfate. However, Km for ATP for N_426_–K is slightly lower which in essence could alter the energetics of the product formation along with sulfate binding for product APS release. Whether APS release is the part where the observed ∼3x higher velocity seen with N_426_–K differs from WT is one of the questions that needs to be answered with reference to higher catalytic efficiency seen with N_426_–K.

## CRediT authorship contribution statement

**K.V. Venkatachalam:** Writing – review & editing, Writing – original draft, Validation, Supervision, Project administration, Methodology, Investigation, Formal analysis, Data curation, Conceptualization. **Dhiraj Sinha:** Visualization, Validation, Software. **Chris Soha:** Validation, Software. **Rudi H. Ettrich:** Visualization, Validation, Supervision, Software, Methodology, Investigation, Formal analysis, Data curation.

## Declaration of competing interest

The authors declare no conflict of Interest at this time.

## Data Availability

Data will be made available on request.
